# Long-term health status as measured by EQ-5D among patients with metastatic breast cancer: comparison of first-line oral S-1 and taxane therapies in the randomized phase III SELECT BC trial

**DOI:** 10.1007/s11136-016-1388-1

**Published:** 2016-08-12

**Authors:** T. Shiroiwa, T. Fukuda, K. Shimozuma, M. Mouri, Y. Hagiwara, H. Doihara, H. Akabane, M. Kashiwaba, T. Watanabe, Y. Ohashi, H. Mukai

**Affiliations:** 1 Department of Health and Welfare Services, National Institute of Public Health, 2-3-6 Minami, Wako, Saitama 351-0197 Japan; 2 Department of Biomedical Sciences, College of Life Sciences, Ritsumeikan University, 1-1-1 Noji-higashi, Kusatsu, Shiga 525-8577 Japan; 3 Kanagawa Academy of Science and Technology (KAST), 3-2-1 Sakado, Takatsu-ku, Kawasaki, Kanagawa 213-0012 Japan; 4 Department of Biostatistics, School of Public Health, The University of Tokyo, 7-3-1 Hongo, Bunkyo-ku, Tokyo, 113-0033 Japan; 5 Breast and Endocrine Surgery Department, Okayama University Hospital, 2-5-1 Shikata-cho, Kita-ku, Okayama, 700-8558 Japan; 6 Department of Surgery, Hokkaido P.W.F.A.C. Asahikawa-Kosei General Hospital, 24-111 Ichijo dori, Asahikawa, Hokkaido 078 8211 Japan; 7 Department of Surgery, Iwate Medical University, 19-1 Uchimaru, Morioka, Iwate 020-8505 Japan; 8 Department of Breast Surgery, Sendai Medical Center, 2-8-8 Miyagino, Miyagino-ku, Sendai, Miyagi 983-8520 Japan; 9 Department of Integrated Science and Engineering, Chuo University, 1-13-27 Kasuga, Bunkyo-ku, Tokyo, 112-8551 Japan; 10 Division of Breast and Medical Oncology, National Cancer Center Hospital East, 6-5-1 Kashiwanoha, Kashiwa, Chiba 277-8577 Japan

**Keywords:** Breast cancer, EQ-5D, Health-related quality of life, Randomized controlled trial, S-1, Taxane

## Abstract

**Purpose:**

The goal of chemotherapy for metastatic breast cancer (MBC) is to prolong survival and maintain health-related quality of life. This study aimed to evaluate long-term health status of patients with MBC who participated in the phase III randomized SELECT BC trial.

**Methods:**

In the SELECT BC trial, patients were randomly allocated to the S-1 or taxane (paclitaxel or docetaxel) arm. Health status was assessed by EQ-5D at pre-treatment, 3 and 6 months after randomization, and every 6 months thereafter to the extent possible. Least square mean scores were assessed to compare EQ-5D index values between groups. Time to deterioration analysis was also performed by defining the minimally important difference of EQ-5D as 0.05 or 0.1.

**Results:**

The number of patients for EQ-5D analysis was 175 and 208 in the taxane and S-1 arms, respectively. Least square mean EQ-5D index values up to 60 months were 0.741 (95 % CI [0.713–0.769]) in the taxane arm and 0.748 [0.722–0.775] in the S-1 arm. The EQ-5D index value during PFS up to 12 months in the S-1 was superior to the corresponding index value in the taxane (0.812 [0.789–0.834] vs. 0.772 [0.751–0.792], *P* = 0.009). Time to deterioration analysis also revealed that S-1 significantly delayed the deterioration of EQ-5D index value during the period before progression (*P* = 0.002 and 0.003).

**Conclusions:**

Our findings suggest that the EQ-5D index value was higher in patients treated with S-1 during first-line chemotherapy. Considering non-inferiority of S-1 in terms of OS, obtained quality-adjusted life years may be greater in the S-1 arm.

## Introduction

Breast cancer has the highest incidence among cancers in women worldwide, with a global age-standardized rate per 100,000 of 51.7 (75.0 in developed countries), corresponding to an increase of 16.6 % from 1990 [1]. Breast cancer is also a major factor that leads to death during the reproductive years [2]. Although diagnosis and treatment of breast cancer have advanced substantially in the past decade, breast cancer is still a common disease that affects the health-related quality of life (HRQOL) of patients and their families, and contributes significantly to healthcare costs.

The goal of chemotherapy for metastatic breast cancer (MBC) is to prolong survival and maintain HRQOL. Taxane and anthracycline are the first choice of chemotherapy for MBC, although these agents can reduce the HRQOL of some patients due to adverse events such as hair loss, peripheral neuropathy, and edema [3, 4]. Patients also frequently need to visit the hospital in order to receive intravenous chemotherapy, which leads to loss of productivity. S-1 [5] is an oral fluoropyrimidine drug for gastric and other cancers. While continuous infusions of 5-FU are known to be more effective than bolus injections for colorectal cancer patients [6], S-1 offers an advantageous and convenient alternative in that the maintenance of therapeutic blood levels of fluorouracil can be achieved with oral tablets alone, without requiring an injection pump.

SELECT BC is a phase III open-label randomized controlled trial (RCT) that compared S-1 with taxane for first-line MBC therapy [7]. The trial demonstrated non-inferiority of S-1 to taxane in overall survival (OS); median OS was 37.2 months in the taxane arm and 35.0 months in the S-1 arm (hazard ratio (HR) 1.05, 95 % CI 0.86–1.27, *P* = 0.015) at a median follow-up of 34.6 months.

SELECT BC also assessed HRQOL as a secondary endpoint using two instruments: the European Organisation for Research and Treatment of Cancer Core Quality of Life Questionnaire C30 (EORTC QLQ-C30) [8] and the three-level version of the EuroQol five-dimensional questionnaire (EQ-5D-3L) [9]. In terms of the primary endpoint, neither treatment is superior to the other; however, our hypothesis expected HRQOL in the S-1 group to be better than in the taxane group. This finding highlights the importance of considering HRQOL in the selection of chemotherapy. In some randomized phase III trials [10–20], the HRQOL of MBC patients was reported using the Functional Assessment of Cancer Therapy (FACT) or EORTC, which are both standard cancer-specific instruments.

In this paper, we report our findings from the long-term data on EQ-5D-3L, which is the most commonly used preference-based measure [21, 22]. EQ-5D-3L index values can be also used to calculate quality-adjusted life years (QALYs) for the economic evaluation of healthcare technologies. As cost-effectiveness is included as a secondary endpoint of this trial, EQ-5D-3L index values will be also used for economic evaluation. In the SELECT BC trial, EQ-5D-3L measurements were continued over a prolonged period of time because measurements could be continued even when the disease progressed, whereas EORTC QLQ-C30 measurements were taken only during the first year. In many studies, measurements are stopped at the pre-determined time point or at disease progression. Thus, the long-term index value is often unknown. Against this backdrop, we report long-term EQ-5D-3L index values of MBC patients measured to the extent possible, i.e., until death.

## Methods

### Study design

In the SELECT BC trial, patients with HER2-negative, hormone-resistant MBC, who were not previously treated with chemotherapy after diagnosis, were randomized at a 1:1 ratio and allocated to the taxane (docetaxel 60–75 mg/m^2^ q3w, paclitaxel 80–100 mg/m^2^ q1w, or paclitaxel 175 mg/m^2^ q3w at the discretion of the treating physician) or S-1 (40–60 mg twice daily based on the patient’s body surface area, for 28 days on and 14 days off) arm. Treatment continued until the disease progressed or more than 4 cycles of S-1 or 6 cycles of taxane were administered.

The enrollment period of the SELECT BC trial was from October 2006 to July 2010, involving 154 institutions. Some of the randomized patients participated in this HRQOL survey. Selection of HRQOL respondents was based on the institution; i.e., some institutions were excluded in advance due to feasibility issues. As institution was a prognostic factor for dynamic allocation, patient background factors were expected to be balanced in both arms.

The study was conducted in accordance with the Ethical Guidelines for Clinical Research of the Japanese Ministry of Health, Labour and Welfare and the Declaration of Helsinki. Written informed consent was obtained from each participant. Approval for the protocol and for any modifications was obtained from an independent ethics committee for each participating site. SELECT BC was prospectively registered with the University Hospital Medical Information Network (UMIN) in Japan (protocol ID C000000416).

### EQ-5D assessment

The EQ-5D comprises five items: “mobility,” “self-care,” “usual activities,” “pain/discomfort,” and “anxiety/depression,” which were assessed at three levels of description. Responses can be converted to an EQ-5D index value using a predetermined algorithm based on societal preferences of the general population.

In the SELECT BC trial, patients were asked to respond to the Japanese version of EQ-5D at baseline, 3, 6, 12 months, and every 6 months thereafter until death or to the extent possible. In general, patients responded to the EQ-5D just before the next cycle of chemotherapy was administered. In this study, a change of 0.05 or 0.1 was considered the minimal important difference (MID) [23–25], which corresponds to the smallest improvement (or deterioration) considered to be worthwhile by the patient.

### Outcome

The predetermined endpoint for EQ-5D is the comparison of longitudinal EQ-5D index values between groups. In addition, explanatory analysis was performed for the following endpoints: time to deterioration by MID during OS and progression-free survival (PFS) and the longitudinal index value after progression and index values before death.

### Statistical analysis

The planned sample population for the HRQOL analysis was approximately 300; this number was not based on a statistical calculation because HRQOL in the SELECT BC trial was not the confirmatory endpoint. Collected responses were converted to EQ-5D index values using the Japanese scoring algorithm [26].

Linear mixed models for repeated measures (MMRM) were applied to compare EQ-5D index values between the two groups. The analysis used all data, including those after disease progression. EQ-5D index values were adjusted by baseline index value, time, and treatment-by-time interaction. Patient was also added to the model as a random variable. Estimates of the least square means for EQ-5D index values and 95 % confidence intervals (CIs) were calculated by each visit and group. An estimated EQ-5D index value during first-line treatment was also calculated, and MMRM was used with progression being censored even if EQ-5D index values were actually measured afterward.

One of the secondary analyses was time to deterioration, with the time assessed using survival analysis. The definition of deterioration was based on the reported MID of EQ-5D: 0.05 or 0.1 relative to the baseline index value. Only the first deterioration was defined as an event. In this analysis, death was also treated as an event because the EQ-5D index value of deceased patients is normally considered to be 0. Another related analysis was time to deterioration during the progression-free period. In this analysis, progression was treated as a competing risk and death as an event. Cumulative incidence function (CIF), Gray’s test [27], and Fine and Gray’s proportional hazard model [28] were applied to these data with competing risk.

Longitudinal index values after progression and before death were also estimated by MMRM. With regard to index values before death, the simple least square mean of index values could potentially have selection bias if the responses of patients with lower EQ-5D index values are more likely to be missing. For example, it may be difficult for end-of-life patients to complete the EQ-5D instrument. To check for potential biases, logistic regression was used to confirm the relationship between “probability of missing at visit 0–6 months before death” and “EQ-5D index value of last observation except at 0–6 months before death.”

These analyses were performed with SAS^®^ 9.4 and R 3.1.0.

## Results

### Patient population

**Fig. 1:**
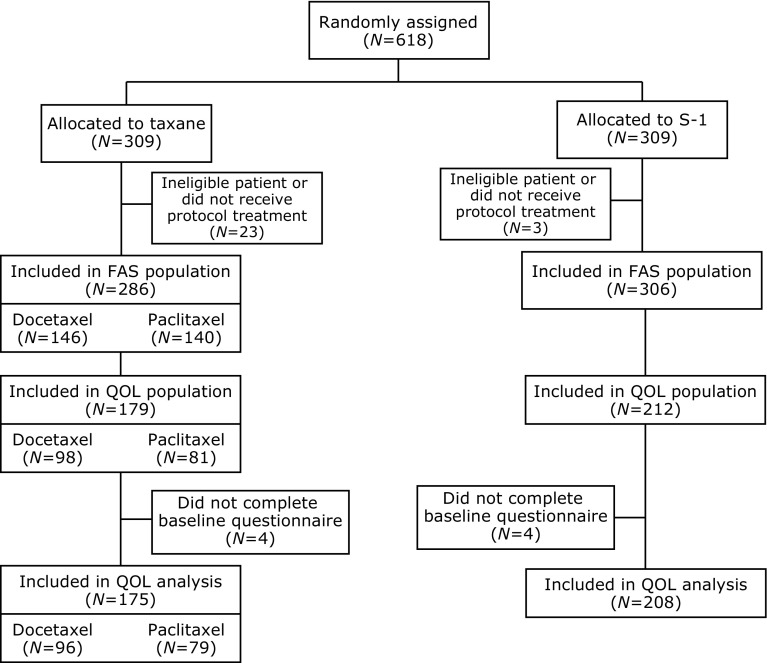
CONSORT diagram

Participants were 618 Japanese MBC patients who were randomly assigned to either the taxane (*n* = 309) or S-1 (*n* = 309) arm. In total, 175 and 208 patients in the taxane and S-1 arms, respectively, were included in the HRQOL analysis. In the taxane arm, 96 patients received docetaxel, and 79 received paclitaxel. The CONSORT diagram for study enrollment is shown in Fig. 1. Baseline characteristics of patients were balanced in the two arms (Table 1).

**Table 1:** Baseline characteristics

	Taxane (*N* = 175)	S-1 (*N* = 208)
Median age (range)	57.0 (33–75)	59.0 (29–75)
Hormone receptor status		
ER positive, PgR positive, or both	127 (72.6)	149 (71.6)
ER negative and PgR negative	45 (25.7)	53 (25.5)
Unknown	3 (1.7)	6 (2.9)
HER2 status		
Negative	162 (92.6)	192 (92.3)
Unknown	13 (7.4)	16 (7.7)
Components of (neo)adjuvant treatment		
Oral fluoropyrimidine	26 (14.9)	22 (10.6)
Taxane	49 (28.0)	61 (29.3)
Endocrine therapy	100 (57.1)	111 (53.4)
Disease-free interval		
≤2 years	34 (19.4)	41 (19.7)
2–5 years	52 (29.7)	66 (31.7)
≥5 years	58 (33.1)	67 (32.2)
No surgery	31 (17.7)	34 (16.3)
Liver metastasis		
Yes	61 (34.9)	78 (37.5)
No	114 (65.1)	130 (62.5)

### EQ-5D completion rates

Longitudinal EQ-5D completion rates are shown in Table 2. Mean duration of the EQ-5D response was 21 months for both groups. Completion rates at 3 months were 88.3 and 83.6 % in the taxane and S-1 arms, respectively, and 71.8 and 77.6 %, respectively, at 12 months. Although the percentage gradually declined with time, more than half of the patients completed the instrument at 60 months (with the exception of 42 and 48 months in the taxane arm).

**Table 2:** EQ-5D completion rate

	Taxane (*N* = 175)	S-1 (*N* = 208)
Baseline	175/175 (100)	208/208 (100)
Month 3	151/171 (88.3)	168/201 (83.6)
Month 6	138/168 (82.1)	146/190 (76.8)
Month 12	107/149 (71.8)	132/170 (77.6)
Month 18	75/126 (59.5)	107/158 (67.7)
Month 24	68/117 (58.1)	93/137 (67.9)
Month 30	51/101 (50.5)	68/110 (61.8)
Month 36	45/90 (50.0)	47/84 (56.0)
Month 42	27/61 (44.3)	31/61 (50.8)
Month 48	18/39 (46.2)	21/37 (56.8)
Month 54	15/23 (65.2)	14/19 (73.7)
Month 60	6/11 (54.5)	8/10 (80.0)

### Longitudinal analysis of EQ-5D index values

**Fig. 2:**
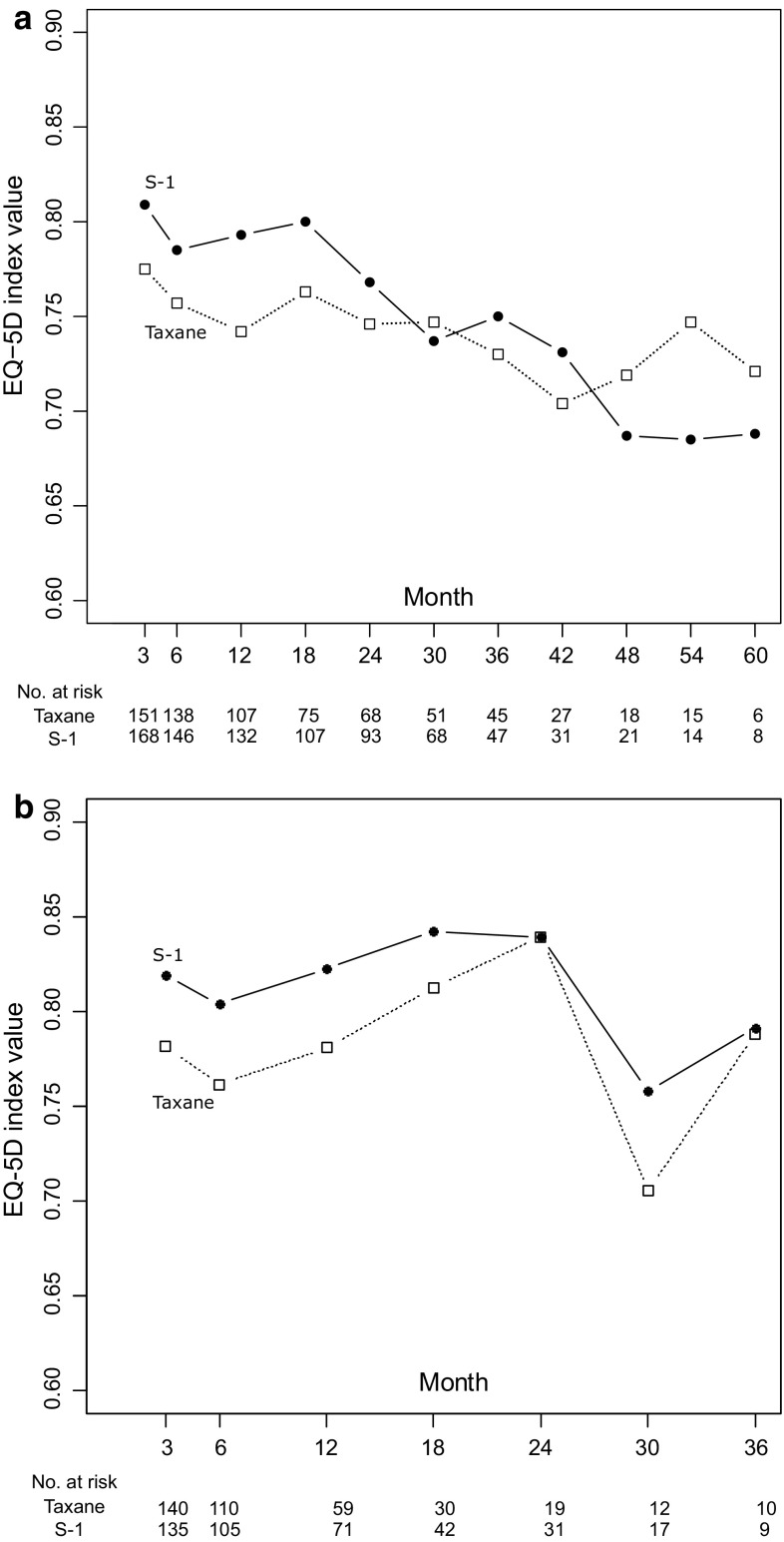
Longitudinal mean and 95% confidence interval of EQ-5D index values. **a** Scores during overall survival. **b** Scores before progression

Estimated EQ-5D least square means and 95 % CI are shown longitudinally in Fig. 2a. Least square means for index values up to 60 months were 0.741 (95 % CI [0.713–0.769]) and 0.748 (95 % CI [0.722–0.775]) in the taxane and S-1 arms, respectively (Table 3). No significant differences in index values were observed between the taxane and S-1 arms up to 60 months (group: *P* = 0.712, interaction of group and time: *P* = 0.691).

**Table 3:** Estimated least square mean EQ-5D index values

	Taxane	S-1
	Least square means [95 % CI]	
(a) During survival		
Up to 60 months	0.741 [0.713–0.769]	0.748 [0.722–0.775]
Up to 36 months	0.750 [0.728–0.772]	0.776 [0.756–0.796]
(b) During progression-free stage		
Up to 36 months	0.781 [0.754–0.809]	0.811 [0.781–0.841]
Up to 12 months	0.772 [0.751–0.792]	0.812 [0.789–0.834]
(c) After progression		
Up to 36 months	0.721 [0.698–0744]	
(d) Before death		
0–6 months before	0.621 [0.584–0.657]	
6–12 months before	0.713 [0.688–0.738]	

Figure 2b shows longitudinal index values and 95 % CI during PFS. This analysis censored responses after progression. EQ-5D index values up to 12 months significantly differed between the two arms (group: *P* = 0.009, interaction of group and time: *P* = 0.966). The period of 12 months approximately corresponds to the duration of chemotherapy, since the median time to treatment failure (TTF) was 8–9 months. Least square means of index values up to 12 months were 0.772 (95 % CI [0.751–0.792]) and 0.812 (95 % CI [0.789–0.834]) in the taxane and S-1 arms, respectively, and index values up to 36 months were 0.781 (95 % CI [0.754–0.809]) and 0.811 (95 % CI [0.781–0.841]), respectively.

### Time to deterioration analysis

**Fig. 3:**
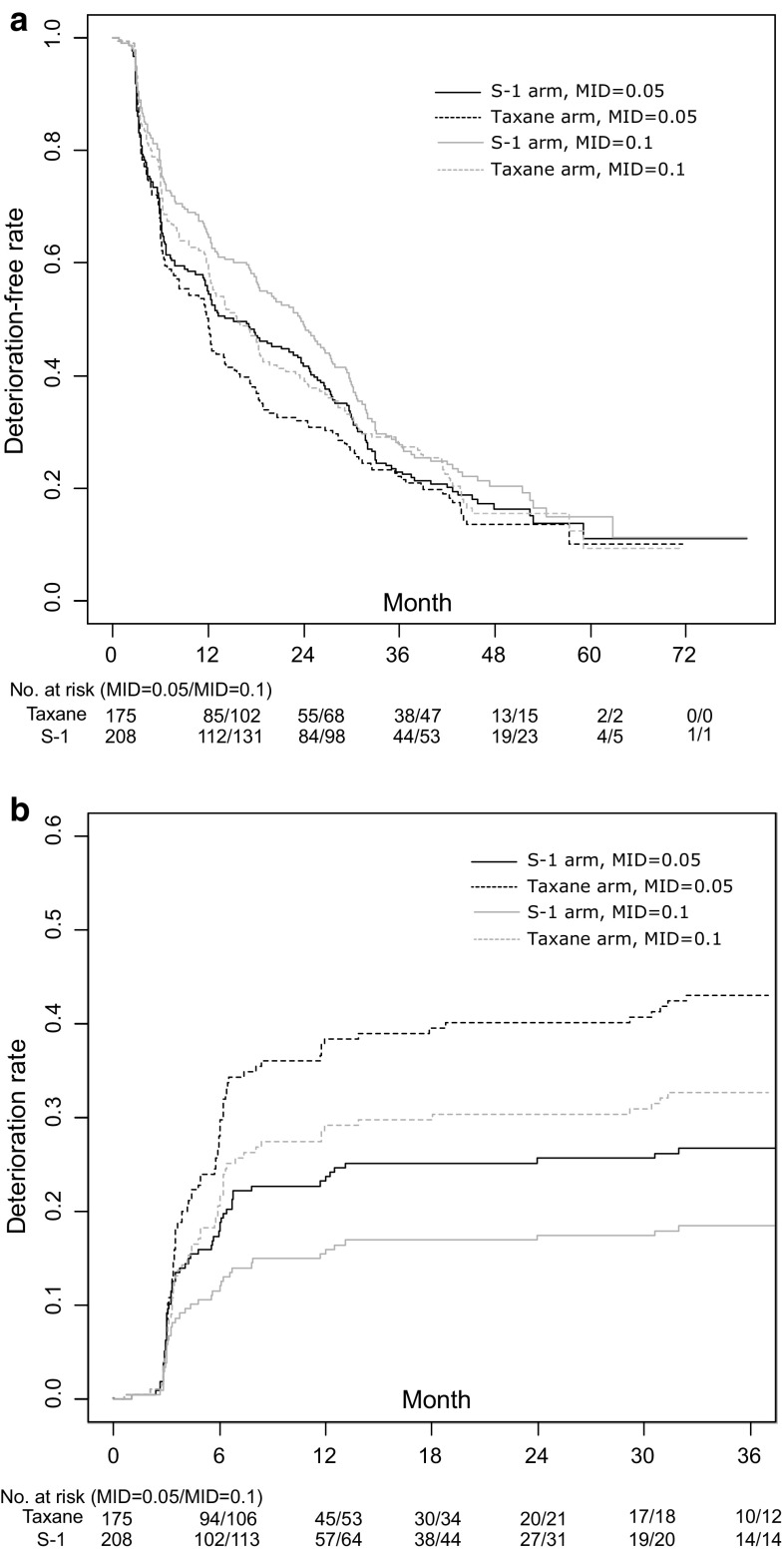
Time to deterioration analysis. **a** Deterioration-free rate during overall survival. **b** Deterioration rate before progression

Figure 3 shows two different time to deterioration analyses, in which 0.05 and 0.1 were used as the MID. First, Fig. 3a shows the deterioration-free rate for all survival periods up to 60 months. Deterioration-free rates did not significantly differ between arms, regardless of the MID. Median time to deterioration was 12.0 and 15.2 months in the taxane and S-1 arms, respectively, when 0.05 was used as the MID. When 0.1 was used as the MID, the time was extended to 15.2 and 23.6 months in the taxane and S-1 arms, respectively. Hazard ratios for the S-1 arm were 0.896 (MID = 0.05, 95 %CI [0.719–1.118], *P* = 0.331) and 0.875 (MID = 0.1, 95 %CI [0.699–1.096], *P* = 0.244).

Deterioration rates during progression-free survival were compared by treating progression as a competing risk. Figure 3b shows CIF of both arms. In this analysis, hazard ratios for the S-1 arm were significantly lower than 1, i.e., 0.580 (MID = 0.05, 95 %CI [0.410–0.820], *P* = 0.002) and 0.536 (MID = 0.1, 95 %CI [0.357–0.804], *P* = 0.003).

### Index values after progression/before death

**Fig. 4:**
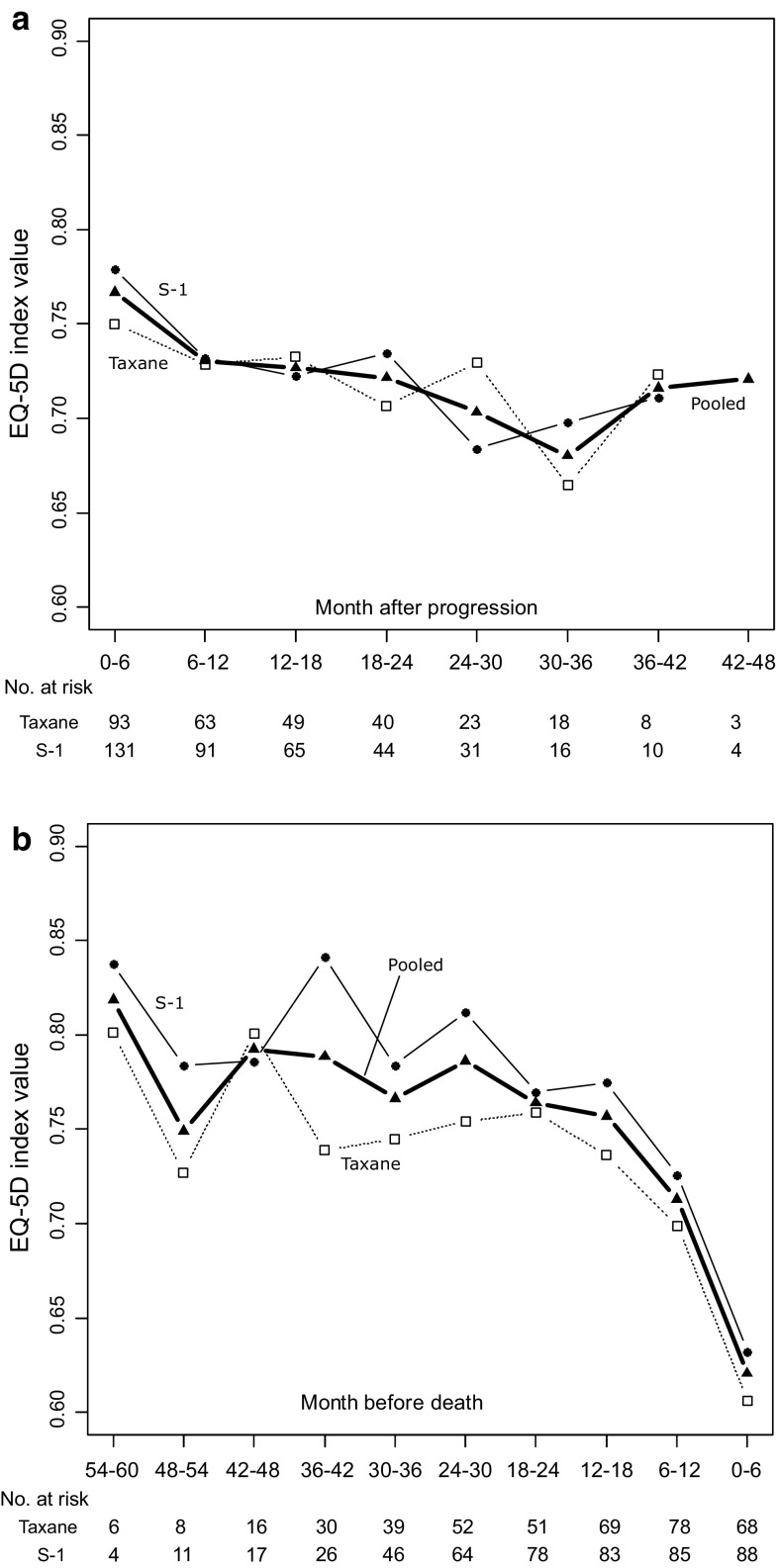
Longitudinal mean EQ-5D index values after progression and before death. **a** Scores after progression. **b** Scores before death

A total of 234 patients (96 in taxane arm and 138 in S-1 arm) had responses after progression. Mean index values were estimated from pooled data (Fig. 4a) because the index values did not significantly differ between arms (group: *P* = 0.877, interaction of group and time: *P* = 0.586). The mean index value after progression was 0.721 (95 %CI [0.698–0744]), which is lower than the index value during first-line treatment.

With respect to index values before death, 256 deaths (116 in taxane arm and 140 in S-1 arm) occurred; 156 responses were collected 0–6 months before death. Mean index values did not significantly differ between arms (Fig. 4b). These results indicate that mean index values decreased gradually as death approached. Logistic analysis revealed that the probability of missing at 0–6 months before death was not significantly correlated with the index value at the last observation (*P* = 0.158). Selection bias in which patients with lower EQ-5D index values tended to drop out was not detected.

## Discussion

HRQOL is an important outcome for patients with MBC. Our study surveyed long-term EQ-5D index values in the phase III randomized SELECT BC trial, which compared S-1 with taxane as first-line treatments for patients with MBC. We found that EQ-5D index values of respondents were higher in the S-1 arm than in the taxane arm, when the analysis was limited to the first 12 months during PFS. Grade 3 or higher adverse events (AE) including edema and sensory neuropathy occurred significantly more frequently in the taxane group, whereas diarrhea, mucositis, and nausea were more frequent in the S-1 group [7]. Different AE patterns might have influenced EQ-5D index values. EQ-5D index values did not differ between the two arms when observations were continued up to 60 months. These results may be due to crossover to S-1 at the time of disease progression or the influence of second-line or subsequent therapy. These results were supported by the results of the time to deterioration analysis. A previous SELECT BC trial report [7] showed that EQ-5D index values were significantly higher in the S-1 arm for 36 months after randomization. In contrast, the present study reports EQ-5D index values over a longer period (60 months), based on more detailed analyses.

EQ-5D index values after progression and before death were also assessed, although these index values did not significantly differ between groups. The SELECT BC trial demonstrated non-inferiority of S-1 to taxane in terms of OS. Considering our EQ-5D estimates, obtained QALYs may be greater in the S-1 arm if the EQ-5D index value of the S-1 arm during chemotherapy is higher.

The completion rate of the instrument was about 85 % at 3 months after randomization, which is the first time point after baseline. The rate at 12 months was about 75 %. The completion rate in the phase III CLEOPATRA trial [17], in which first-line MBC patients were randomly allocated to pertuzumab plus existing treatment (trastuzumab plus docetaxel) or placebo plus existing treatment, was 83.9 % in the placebo arm and 79.9 % in the pertuzumab arm at 9 weeks. Rates decreased to 76.5 and 81.1 % at 54 weeks (approximately 12 months), respectively. In the randomized JGOG3016 trial [29] for stage II–IV ovarian cancer conducted in Japan, completion rates were 74.5 % for conventional treatment and 73.0 % in the dose-dense chemotherapy arm at the third cycle (approximately 9 weeks) and 74.2 and 71.6 % at 12 months after randomization. When considering these rates, ours were similar to those of other randomized phase III studies.

We used both the MMRM and the time to deterioration analysis to compare EQ-5D index values between groups. Many recent studies have begun to use time to deterioration analysis. While the analysis may be more widely accepted, the results depend on various settings, such as the size of MID and the handling of other events. Indeed, as shown in Fig. 3, the size of MID clearly influenced the results. Results also differed depending on whether death or progression was considered an event or was censored, or neither. While our analysis treated death as an event, if it was censored in the sensitivity analysis, the Kaplan–Meier curves and median time to deterioration would likely have changed. Standardization of time to deterioration analysis is reportedly needed to improve transparency and comparability [30].

This study has some limitations. First, the SELECT BC trial was an open-label trial that compared oral and intravenous therapies. Thus, patients could readily determine which arm they were allocated to. Some patients participated in the trial in order to receive oral chemotherapy; if they were not allocated to the preferred arm, their disappointment might have led to decreased EQ-5D index values. Second, our analysis did not include missing index values at each time point. MMRM can be applied to data under the assumption of missing at random, which means that a missing index value can be predicted by the observed index value. However, if, for example, the missing index value depended on the value after drop out, the estimated index value by MMRM may be biased. Finally, in this study, EQ-5D was measured at intervals of 3–6 months, which might have been too long to capture a temporal deterioration of HRQOL (e.g., AE caused by chemotherapy), given that EQ-5D asks respondents about their momentary health state.

In conclusion, our study supports that the EQ-5D index value for the S-1 arm is higher during first-line chemotherapy. We also demonstrated that EQ-5D index values are similar between patients treated with S-1 and those treated with taxane over a prolonged period of time. In addition, we reported on EQ-5D index values after progression and before death. These data can contribute not only to economic evaluations, but also to the selection of treatment for first-line MBC patients based on individual preferences.
